# Novel RNA Extraction Method for Dual RNA-seq Analysis of Pathogen and Host in the Early Stages of *Yersinia pestis* Pulmonary Infection

**DOI:** 10.3390/microorganisms9102166

**Published:** 2021-10-18

**Authors:** Ofir Israeli, Inbar Cohen-Gihon, Moshe Aftalion, David Gur, Yaron Vagima, Ayelet Zauberman, Yinon Levy, Anat Zvi, Theodor Chitlaru, Emanuelle Mamroud, Avital Tidhar

**Affiliations:** Department of Biochemistry and Molecular Genetics, Israel Institute for Biological Research, Ness-Ziona 74100, Israel; Ofiri@iibr.gov.il (O.I.); Inbarg@iibr.gov.il (I.C.-G.); moshea@iibr.gov.il (M.A.); gurd@iibr.gov.il (D.G.); yaronv@iibr.gov.il (Y.V.); ayeletz@iibr.gov.il (A.Z.); yinonl@iibr.gov.il (Y.L.); Anatz@iibr.gov.il (A.Z.); emmym@iibr.gov.il (E.M.)

**Keywords:** dual RNA-seq, pneumonic plague, *Y. pestis*, mouse model, infection

## Abstract

Pneumonic plague, caused by *Yersinia pestis,* is a rapidly progressing lethal infection. The various phases of pneumonic plague are yet to be fully understood. A well-established way to address the pathology of infectious diseases in general, and pneumonic plague in particular, is to conduct concomitant transcriptomic analysis of the bacteria and the host. The analysis of dual RNA by RNA sequencing technology is challenging, due the difficulties of extracting bacterial RNA, which is overwhelmingly outnumbered by the host RNA, especially at the critical early time points post-infection (prior to 48 h). Here, we describe a novel technique that employed the infusion of an RNA preserving reagent (RNAlater) into the lungs of the animals, through the trachea, under deep anesthesia. This method enabled the isolation of stable dual mRNA from the lungs of mice infected with *Y. pestis*, as early as 24 h post-infection. The RNA was used for transcriptomic analysis, which provided a comprehensive gene expression profile of both the host and the pathogen.

## 1. Introduction

Bacteria have successfully evolved sophisticated mechanisms that allow them to sense, cope with, and adapt to varying conditions in their immediate surroundings. Often, these mechanisms are essential for the manifestation of virulence in bacterial pathogens [1]. The rapid detection of extracellular signals, such as the concentrations of nutrients, ion sources, temperature, stress conditions, and the presence of host immune cells, influences transcriptional regulatory systems, leading to physiological and morphological changes that enable the pathogen to survive within hostile environments, such as those encountered in the host during infection [2,3]. The Gram-negative pathogen *Yersinia pestis*, the causative agent of plague, has been responsible, in the past, for major pandemics, which caused the death of over 200 million people and still represent a significant public health concern in endemic regions, particularly in the third world. According to the report of the World Health Organization (WHO), since 2010, there have been hundreds of people that have died of plague. In 2017, 2348 cases of plague were reported in Madagascar, including 1791 cases of pneumonic plague, which caused 200 deaths. In the wild, plague mostly affects rodent populations, by flea bites. In some cases, *Yersinia pestis* afflicts populated areas and may manifest in humans as bubonic plague; although, in some cases, it causes primary septicemia, without detectable characteristic bubos. Secondary pneumonia may evolve from bubonic and septicemic plague [4]. In the case of pneumonic plague, the disease becomes highly contagious, as the bacteria are transmissible by air, causing deadly primary pneumonic presentations. The ability of *Y*. *pestis* to invade the mammalian host, colonize internal organs, and overcome the immune response is attributed to the combined activities of multiple virulence pathways that are activated in a timely manner during infection, in response to the host *milieu* signals [5,6]. Some of these pathways appear to be absolutely necessary for bacterial pathogenesis in animal models; therefore, their inspection directly in the host, rather than in vitro, is essential for deciphering their role. The ability to obtain a complete RNA-seq transcriptome profile of bacteria in different physiological states, using high-throughput sequencing, has notably enhanced the ability to understand dynamic biological processes [7], including those that enable bacterial pathogens to successfully colonize their hosts [8]. The extraction of bacterial RNA from infected lungs in the early stages of pneumonic plague, which are critical for the subsequent evolution of the disease, is challenging and often unsuccessful, due to the low number of bacterial cells and the high content of host RNA. Therefore, most of the reported studies have employed dual host/pathogen RNA-seq analysis, using samples collected at advanced stages of the infection [3,6,9,10], when the early critical molecular events involved in the onset of the infection are often undetectable. In this study, we present a novel method for isolating bacterial RNA from the lungs of infected mice during the early stages of pneumonic plague, enabling a comprehensive dual RNA-seq analysis for the subsequent study of host–pathogen interactions. While the present report documents the implementation of the method for *Yersinia* infection, it may be applied to study other pulmonary infections.

## 2. Materials and Methods

### 2.1. Bacterial Strains

The bacterial strain examined in this study was the virulent *Y. pestis* strain Kimberley53 (Kim53) [11]. The bacteria were used for intranasal infections, as described previously [12]. Briefly, *Y. pestis* bacteria were grown on BHIA plates (BD, Franklin Lakes, MD, USA) for 48 h at 28 °C, and several colonies were suspended in HIB (BD, Franklin Lakes, MD, USA) supplemented with 0.2% (+) xylose and 2.5 mM CaCl_2_ (Sigma-Aldrich, St. Louis, MO, USA) and incubated overnight at 28 °C. The culture was diluted in saline solution to achieve the required infection dose.

### 2.2. Experimental Animals

All animal experiments were approved by the IIBR Committee for Animal Research (protocols M-63-2017 and M-31-2017). The experimental animals were handled according to the National Research Council 1996 Guide for the Care and Use of Laboratory Animals and regulations of the IIBR Animal Use Committee.

Female OF1 mice (5–6 weeks old) were purchased from Charles River (UK). Prior to infection, mice were anaesthetized by intraperitoneal injection of a mixture of 0.5% ketamine HCl and 0.1% xylazine, then subjected to intranasal infection with 35 μL/mouse of the bacterial suspension. Under these conditions, the LD_50_ of *Y. pestis* Kim53 is ~550 CFU [11,12]. Mice were infected with 1000 LD_50_ (550,000 CFU). Of note, 24 hpi the bacterial load in the lungs was 4 × 10^7^ CFU/animal, and 48 hpi, the load was 1.8 × 10^9^ CFU/animal. The infectious dose was verified by plating diluted bacterial suspensions onto BHIA plates. To collect lung tissue, mice were anesthetized with 0.1 mL (10% ketamine (100 mg/kg) + 2% xylazine (10 mg/kg))/10 g body weight.

### 2.3. RNA Extraction from Spiked Tissue

The lungs of naive mice were mashed in 1 mL of RLT buffer (RNeasy Mini Kit, Qiagen, Hilden, Germany) and spiked with 10^6^ or 10^7^ CFU of *Y. pestis* bacteria. RNA was extracted to 50% of the initial volume using the RNeasy Mini Kit (Qiagen, Hilden, Germany).

### 2.4. RNA Extraction from Infected Mice Lungs

**Figure 1:**
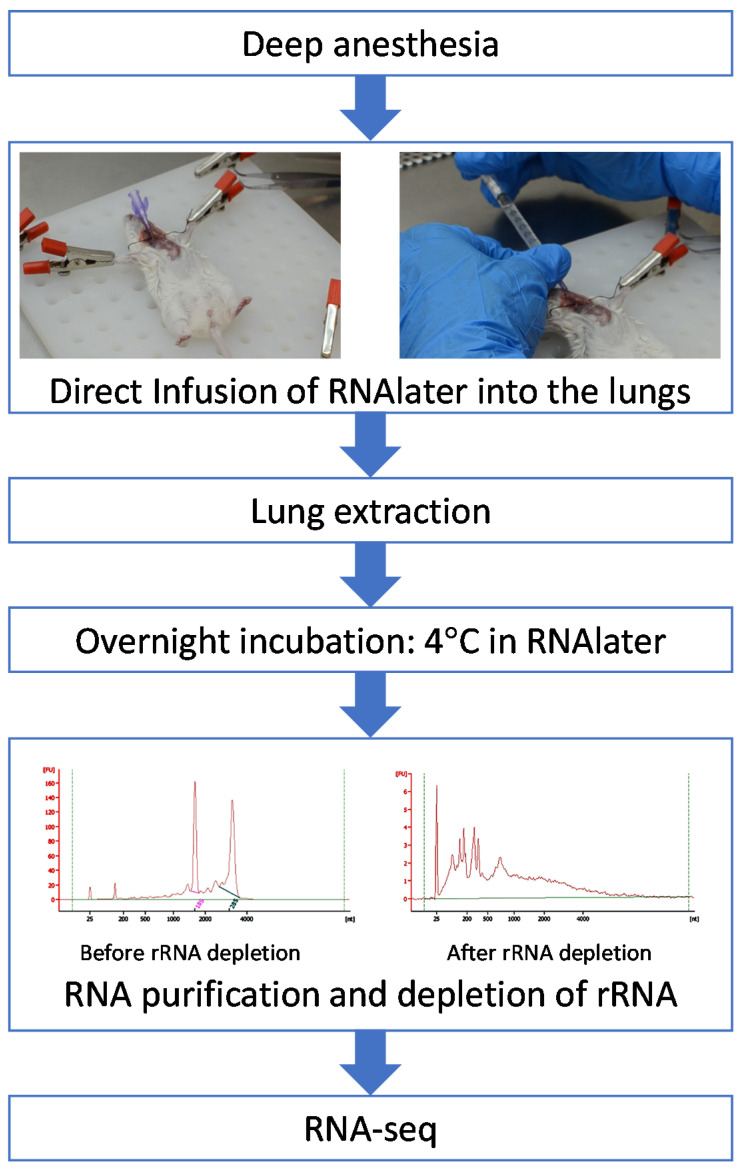
Flow chart of the method, from murine lung collection for RNA extraction to RNA-seq analysis. The upper panels illustrate the infusion of RNAlater into the mouse lungs through the trachea under deep anesthesia. The rRNA depletion step shown as follows: On the left, RIN analysis of high-quality total RNA (RIN = 8.7) extracted from mouse lungs. On the right, the same sample after rRNA depletion. Note the enrichment of the mRNA fraction and the virtually complete removal of the mammalian rRNAs 18S and 28S rRNAs (in pink and green on the left panel) and the bacterial 16S and 23S rRNAs.

We implemented a novel method that employed in situ infusion of the RNA preserving reagent RNAlater (Thermo Fisher Scientific, Waltham, MA, USA) into the lungs of the animals through the trachea under deep anesthesia. A puncture tracheotomy technique [13,14] enabled infusion of 1 mL of RNAlater into the lungs through the trachea using a syringe attached to an IV catheter (Figure 1, top panel). After infusion of RNAlater, the thoracic cavity was dissected, the lungs were removed and immediately washed in PBS by immersion and subsequently placed in a 50 mL test tube containing 25 mL RNAlater. The entire lungs were incubated overnight at 4 °C with continuous agitation and subsequently mashed through a 70 µm cell strainer (Falcon, NY, USA) with a 3 mL syringe plunger into 1 mL RLT buffer and 1 mL of 70% ethanol. The tissue extract was mixed by vortex for a few seconds and divided into two identical volumes, each of which was transferred to an RNA preparation column (each sample was performed in two similar replicates). RNA was extracted according to the manufacturer’s instructions (RNeasy Mini Kit, Qiagen, Hilden, Germany) and eluted with 50 µL of RNase-free water.

### 2.5. mRNA Enrichment

The RNA samples were subjected to rRNA removal using the Ribo-Zero Gold rRNA Removal Kit (Illumina, San Diego, CA, USA) according to the manufacturer’s instructions. The RNA quality was evaluated by RIN (RNA integrity number) analysis with the BioAnalyzer device (Agilent, Santa Clara, CA, USA) using the RNA high-sensitivity chip.

### 2.6. Bioinformatic Analysis

RNA-seq was performed at the JP Sulzberger Columbia Genome Center (New York, NY, USA). Following RiboZero rRNA depletion, libraries were constructed using the TruSeq RNA library preparation kit (Illumina) without the poly A pulldown step. Whole transcriptome sequencing (total RNA-seq) using an Illumina HiSeq was performed producing paired-end 100 nt reads. Quality control was performed using fastQC version 0.11.5, checking for per-base sequence quality, per-sequence quality scores, and adapter content. All reads of the bacterial transcriptome were mapped to the reference genome *Y. pestis* CO92, available in the NCBI database (accession numbers: NC_003143 (chromosome), NC_003131 (pCD1), NC_003132 (pPCP1), NC_003134 (pMT1)). This genome, also belonging to the *Orientalis* biovar (as the Kim 53 strain used for infection) served as a reference for *Y pestis* genomic sequence analysis. Mapping was performed using Bowtie 2, version 2.2.0 [15], with default parameters. The expression level of each gene was determined using HTSeq, version 0.6.1 [16], using GFF *Y. pestis* CO92 feature file, downloaded from the NCBI database.

The reproducibility of the biological replicates was evaluated by principal component analysis (PCA) using the R package DESeq2, version 1.26.0 (13). Similarity matrix and hierarchical clustering were generated using the R packages gplots and RColorBrewer.

The generated data will enable the dual transcriptome to be profiled and insights to be gained into the processes involved during *Y. pestis* infection. The RNA-seq raw data were deposited in the NCBI public domain and are available under accession number GSE181680.

To evaluate the consistency of the method and the compatibility of the sequences for further analysis, the various samples were examined using heatmap and principal component analysis (PCA).

## 3. Results

To evaluate the efficiency of extracting dual RNA from the lungs of plague-infected mice, by conventional methods, the mashed lungs of naïve mice were spiked with *Y. pestis.* The tissue embedment was used for RNA extraction, and approximately 50 ng/µL of RNA could be extracted from the tissue spiked with 10^6^ CFU, and 200 ng/µL from the tissue spiked with 10^7^ CFU. The RIN (RNA integrity number) of the RNA samples was >8 for all the samples, indicating that the RNA quality was adequate for downstream RNA-seq analysis (data not shown). Next, RNA was extracted from the dissected lungs of the *Y. pestis*-infected mice, under the same conditions. The *Y. pestis* load in the lungs at 24 h post-infection (hpi) was 10^7^ CFU, which was shown to be adequate for RNA extraction (see above). Nevertheless, the initial attempts to obtain high-quality RNA from the early time points after infection (24 hpi), in a sufficient quantity, were unsuccessful by us and others (for example, see [6]). This complication was reflected by a low and difficult-to-quantify RIN value (see Supplementary Figure S1), as well by the failure to obtain NGS reads for further RNAseq analysis. Of note, this constraint is acknowledged as a major obstacle in the analysis of host/pathogen dual RNA in the early stages post-infection [17,18].

This limitation, probably owing to the overwhelming excess of host RNA and the poor stability of the bacterial RNA, could be alleviated by the implementation of the novel protocol, which includes pre-infusion of the lungs with an RNA preserving reagent. The protocol enabled the extraction of high-quality RNA (Table 1). The samples extracted at 24 hpi contained a sufficient amount of RNA (27–56 ng/µL), which exhibited RIN values >8. At 48 hpi, 27–33 ng/µL of RNA was obtained. Although the integrity of each sample was high, without notable RNA degradation, due to the dual content of the samples, lower RIN values were observed (owing to the high value of 16S and 23S contributed by the bacterial rRNA, Table 1). The enrichment of mRNA in the samples enabled improvement of the percentage of mRNA, from 2–3% to >90% (Table 1 and Figure 1, bottom panel). The mRNA was analyzed by RNA-seq at the Sulzberger Genome Center (Columbia University, New York, NY, USA), yielding more than 60 million 100-bp reads per sample, composed of 2.1 to 4.6 million (for mice sacrificed at 24 hpi), and 4.5 to 10.2 million (for mice sacrificed at 48 hpi) *Y. pestis* reads/sample (Table 1), as well as over 40 million mouse reads/sample (Table 1).

**Figure 2:**
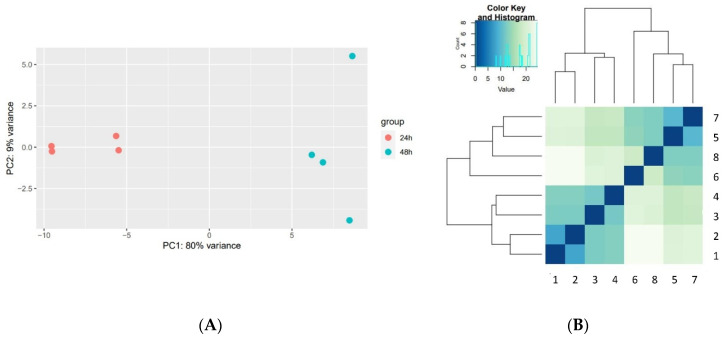
PCA and heatmap plots of dual RNA samples collected 24 and 48 hpi. (**A**) PCA plot and (**B**) distance heatmap. Mice infected with 1000 LD_50_ of *Y. pestis* Kimberly53 strain were sacrificed, lungs were collected and used for RNA extraction. Samples were sequenced individually and the results were used for PCA plot and for heatmap showing gene expression changes induced after 24 or 48 hpi. (**A**) Orange circles—24 hpi; blue circles—48 hpi. (**B**) Samples numbers correspond to those in Table 1. Scale bar in (**B**) shows z-score of normalized read counts (CPM).

Cluster analysis, including PCA and corroborated by the heatmap depiction of highly modulated genes, evaluated possible pairwise structural dissimilarities between the datasets generated by the inspection of similar samples. PCA provides information pertaining to the interrelationship among variables, and the heatmap depicts a two-way trait and accession classification. The results depicted in Figure 2 substantiate the robustness of the method, as they clearly demonstrate the similarity between independent samples prepared under the same conditions, and the difference between the samples collected at various time points (24 vs. 48 hpi). The method enabled, for the first time, transcriptomic analysis of plague in the early stages after infection (24 hpi) and a comparison of the early transcriptomic profile to that of a later stage after infection (48 hpi). The gene expression RNA-seq-generated data (17 datasets) were made publicly available in the NCBI repository (see Material and Methods and Data Availability Statement). The data include the following: (i) gene expression profiling of bacteria grown in vitro at 28 or 37 °C (in duplicates, to serve as a control for the in vivo, specifically induced bacterial genes), (ii) gene expression profile of murine lungs (two naïve mice, in duplicates), and (iii) dual murine and bacterial gene expression data of lungs collected 1 hpi (three mice), 24 hpi (four mice), and 48 hpi (four mice). The considerable difference between the samples collected at 24 and at 48 hpi (Figure 2) is in line with the known evolution of the disease. Further analysis of these dual transcriptome datasets will facilitate gaining insights into the processes involved in *Y. pestis* infection.

## 4. Conclusions

Previous attempts to extract RNA from the airways of infected animals, for in vivo dual pathogen–host RNA-seq analysis, generated inferior results [3,6,19]. The extraction of sufficient amounts of bacterial RNA in the early stages post-infection is particularly challenging. Often, the RNA-seq profile of the samples does not entail sufficient sequencing reads for accurate downstream gene expression analysis. In most of the cases, this is mostly the result of a low pathogen load in the respiratory system and an overwhelming prevalence of host RNA. Furthermore, this limitation may be the consequence of RNAse activities that are possibly released from the host cells in the process of tissue collection. In this brief report, we document a novel procedure that enabled the extraction of high-quality dual mRNA, in sufficient amounts, from bacteria-infected lungs in the early stages of pneumonic infection, enabling RNA-seq transcriptomic analysis. According to this novel protocol, an RNA preserving reagent is directly infused through the trachea to the lungs, resulting in an immediate and abrupt cessation of all biological processes and, consequently, the preservation of RNA in an intact conformation that is amenable for analysis. Of note, the “classical” procedure (including dissection, extraction of lungs, immersion in an RNA preserving solution, homogenization of tissue, RNA preparation Trizol-based protocol) is not sufficiently rapid for preventing RNAse activity, which is substantial after sacrificing the animal, and before the immersion of the extracted lungs in the RNA preserving solution. In addition, it is conceivable that the direct and active infusion of the RNA preserving solution into the lungs, prior to sacrificing the animals (by means of intra-tracheal injection, see Figure 1), is beneficial, in the sense that the solution reaches the intra-pulmonary compartments significantly more efficiently than immersion of the lungs. The distinct clustering of the replicate samples extracted at different time points, emerging from the heatmap and PCA, demonstrate the robustness of the method. Actually, the distinct clustering is characteristic of the progress of the respiratory plague disease, which is manifested by a typical two-stage course, entailing a sharp change in the host response between 24 and 48 hpi [6,14]. The high-quality RNA-seq data generated by this novel dual RNA extraction approach, from the lungs of animals infected with *Y. pestis*, were made publicly available for further identification of the bacterial process and host responses occurring in the course of the infection. While the protocol was demonstrated in the murine pneumonic plague model, it may be applied to other pneumonic bacterial infections.

## Figures and Tables

**Table 1:** Dual RNA samples analyzed in this study. The experiment included four mice at each time point. The RNA-seq profiles of the mice obtained using these 8 samples were deposited in the NCBI repository as individual datasets (see Materials and Methods).

	Sample No.	RIN of Sample	RNA Concentration (ng/µL)		Total RNA Reads	Mouse RNA Reads	Bacterial RNA Reads
			Before Depletion	After Depletion			
**Whole lung** **24 hpi**	1	8.7	52	1.3	60,325,276	42,909,562 (71.13%)	3,889,876 (6.45%)
	2	8.4	56	1.95	82,104,539	58,227,020 (70.92%)	4,637,343 (5.65%)
	3	8	27	1.7	51,954,957	34,715,948 (66.82%)	2,306,038 (4.44%)
	4	8.7	34	0.5	61,118,291	45,386,746 (74.26%)	2,101,363 (3.44%)
**Whole lung** **48 hpi**	5	6.7	30	3.3	52,065,573	29,282,281 (56.24%)	10,214,742 (19.62%)
	6	6.5	20	2.4	49,791,084	32,695,959 (65.67%)	4,724,302 (9.49%)
	7	6.7	27	2.6	69,861,786	41,193,569 (58.96%)	6,297,704 (9.01%)
	8	6.6	33	0.66	65,972,823	39,385,308 (59.7%)	4,497,619 (6.82%)

## Data Availability

The RNA-seq dual-gene expression datasets generated in the transcriptomic study were submitted at the NCBI depository and are publicly available under the accession number GSE181680 (project title: RNA-seq analysis of pathogen and host in the early stages of plague pulmonary infection using a novel dual RNA extraction method). The project includes 17 datasets, representing data obtained by multiple reiterations of various collection time points in the course of infection.

## Supplementary Materials

The following are available online at www.mdpi.com/10.3390/microorganisms9102166/s1, Figure S1: Raw RIN (RNA Integrity Number) profiles of 3 replicated samples (samples1, 2 and 3, as indicated) of infected murine lungs harvested 24 h post onset of infection.
