# Supplementary material for: Novel RNA Extraction Method for Dual RNA-seq Analysis of Pathogen and Host in the Early Stages of Yersinia pestis Pulmonary Infection
Source: Microorganisms. 2021 Oct 18;9(10):2166. doi: 10.3390/microorganisms9102166 (PMC8539884; doi:10.3390/microorganisms9102166)
Supplement: Supplementary file 1 [file microorganisms-09-02166-s001.zip › microorganisms-1362200-supplementary.pdf]

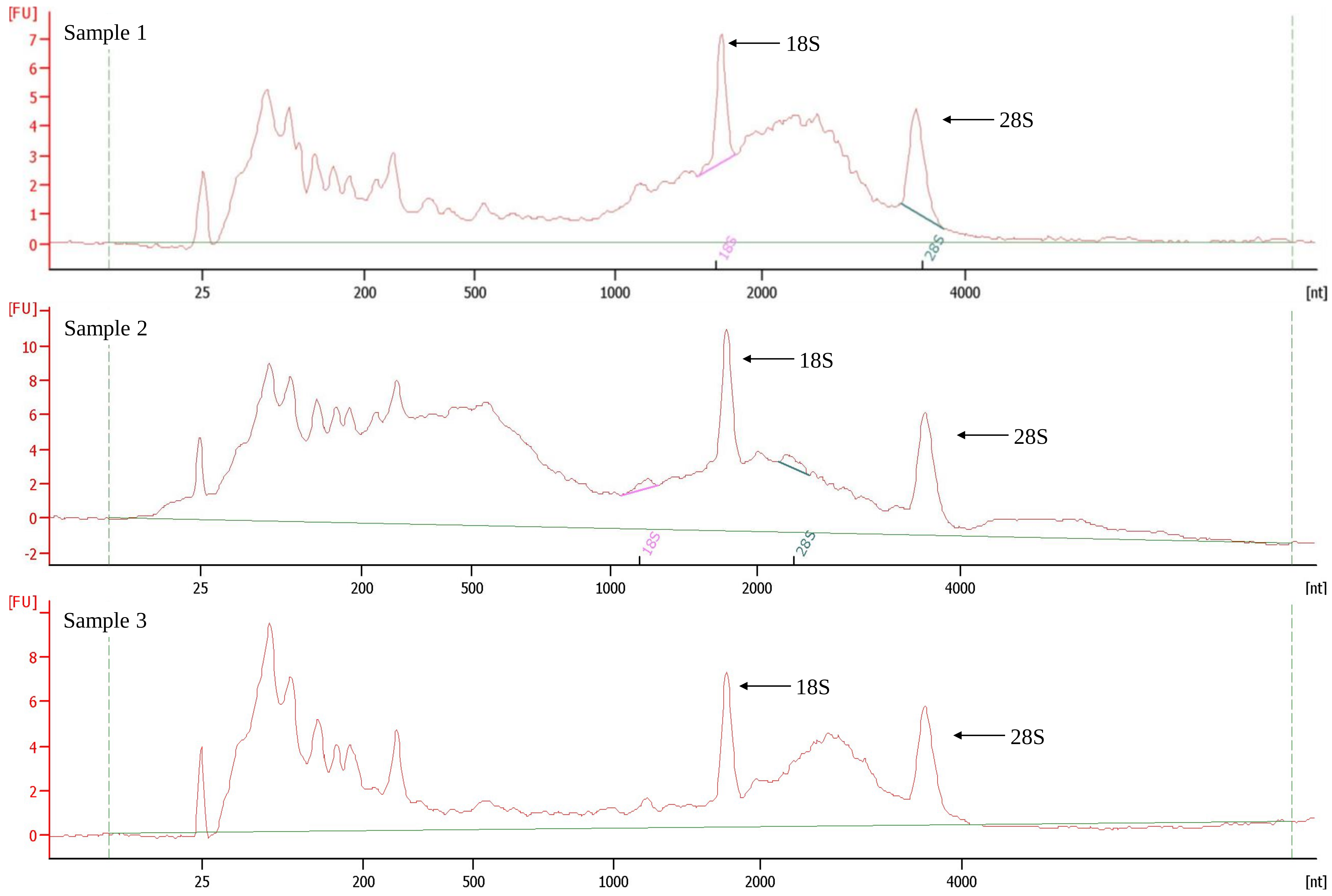


**Supplementary Figure S1** Raw RIN (RNA Integrity Number) profiles of 3 replicated samples (samples1, 2 and 3, as indicated) of infected murine lungs harvested 24 hours post onset of infection.
RNA was obtained employing the "classical" extraction method consisting of immersion of the lungs into an RNA preserving reagent. Note the significant degradation of the RNA in the three replicated samples. Arrows indicate peaks corresponding to the 18S and 28S ribosomal RNA. The calculated RIN values were: Sample 1: 3.6; 2 and 3: under quantification limit. This low quality of the samples, conceivably precludes sufficient representation of the bacterial RNA for subsequent analysis (see text).
